# Liquid Biopsy in Clear Cell Renal Cell Carcinoma: Diagnostic Potential of Urinary miRNAs

**DOI:** 10.3390/cancers18020285

**Published:** 2026-01-16

**Authors:** Giacomo Vannuccini, Alessio Paladini, Matteo Mearini, Francesca Cocci, Giuseppe Giardino, Paolo Mangione, Vincenza Maulà, Daniele Mirra, Ettore Mearini, Giovanni Cochetti

**Affiliations:** Urology Clinic, Department of Medicine and Surgery, Santa Maria della Misericordia Hospital, University of Perugia, 06129 Perugia, Italy; alessio.paladini@ospedale.perugia.it (A.P.); francesca.cocci@specializzandi.unipg.it (F.C.); giuseppe.giardino@specializzandi.unipg.it (G.G.); paolo.mangione@specializzandi.unipg.it (P.M.); vincenza.maula@unipg.it (V.M.); daniele.mirra@specializzandi.unipg.it (D.M.); ettore.mearini@unipg.it (E.M.); giovanni.cochetti@unipg.it (G.C.)

**Keywords:** urinary microRNAs (miRNAs), clear cell renal cell carcinoma (ccRCC), liquid biopsy, non-coding RNAs, renal cancer biomarkers, urinary biomarkers

## Abstract

Background: Clear cell renal cell carcinoma (ccRCC) is the most prevalent kidney cancer subtype and, in most cases, it is incidentally diagnosed, as early-stage disease is often asymptomatic. Therefore, the identification of stable, noninvasive biomarkers is a major unmet clinical need. Urinary microRNAs (miRNAs) have emerged as promising candidates since they are extraordinarily stable in urine and show a close relationship with tumour biology. Methods: In this study, urinary expression levels of five miRNAs (*miR-15a*, *miR-15b*, *miR-16*, *miR-210*, and *miR-let-7b*) were analysed in RCC patients before surgery, 5 days after, and one month after surgery, and compared to healthy controls. Results: Non-parametric analyses revealed significant postoperative decreases for *miR-15a* (*p* = 0.002), *miR-16* (*p* = 0.025), *miR-210* (*p* = 0.030), and in the overall *miRNA Sum* (*p* = 0.002), suggesting that these miRNAs are directly linked to tumour presence. In the comparison between preoperative and one-month postoperative samples, *miR-let-7b* (*p* = 0.049) and the global *miRNA Sum* (*p* = 0.037) remained significantly reduced after intervention, indicating a partial normalisation of urinary miRNA profiles. Correlation analyses demonstrated positive associations between specific miRNAs and clinical parameters such as age, ischemia time, and surgical time, reinforcing their potential relevance to tumour biology and treatment response. Conclusions: These findings support urinary miRNAs as promising, minimally invasive biomarkers for ccRCC diagnosis and postoperative monitoring.

## Simple Summary

Clear cell renal cell carcinoma is the most common form of kidney cancer and is often discovered incidentally because early disease rarely causes symptoms. This creates an urgent need for non-invasive biomarkers that can help in tumour detection and monitor patients after surgery. MicroRNAs in urine are promising candidates because they are stable and reflect tumour activity. In this study, we measured five urinary microRNAs in urine of patients before surgery, shortly after surgery, and one month later, and compared the results with those from healthy individuals. Several microRNAs decreased after tumour removal, suggesting that their levels are closely related to the presence of cancer. Some microRNAs were also associated with clinical features, supporting their biological relevance. These findings indicate that urinary microRNAs may serve as useful, minimally invasive biomarkers for diagnosing clear cell renal cell carcinoma and for monitoring patients after treatment.

## 1. Introduction

Renal cell carcinoma (RCC) arises predominantly from the proximal tubular epithe-lium and represents a highly heterogeneous group of tumours with a strong tendency to metastasize, often even before diagnosis [1]. More than 16 histological subtypes of RCC have been described, among which clear cell RCC (ccRCC) is the most prevalent, accounting for about 70–80% of cases, followed by papillary RCC (10–15%), chromophobe RCC (approximately 5%), and collecting duct RCC (<1%) [2]. The incidence and mortality of RCC are steadily increasing, largely attributed to the widespread use of abdominal imaging in routine clinical practice, which has increased the number of incidental tumour detections. Despite advances in imaging and therapeutic strategies, the prognosis of metastatic RCC (mRCC) remains poor, with survival ranging from a few months to approximately five years, depending on disease aggressiveness [3]. In addition, the rising detection of small renal masses poses a significant clinical challenge, as it is often difficult to distinguish malignant lesions from benign ones, such as oncocytomas and papillary tumours. Histological confirmation through tissue biopsy remains the current diagnostic gold standard; however, this approach presents several limitations. Tissue biopsies are invasive procedures associated with potential complications such as bleeding, infection, and insufficient sampling. A further concern is the possibility of tumour seeding, which may contribute to disease progression. In addition, some patients are not suitable candidates for biopsy due to tumour inaccessibility or comorbidities that increase procedural risks [4]. The intrinsic intratumoural heterogeneity of ccRCC further limits the diagnostic accuracy of a single biopsy, as it may fail to capture the full molecular and histological complexity of the tumour [5,6].

Given these limitations, there is a growing interest in developing non-invasive diagnostic approaches. Liquid biopsies have emerged as a promising tool for RCC assessment, offering the possibility of detecting and monitoring cancer through the analysis of biomarkers in different body fluids [4]. This approach relies on the analysis of circulating tumour DNA (ctDNA), circulating tumour cells (CTCs), or other biomarkers in body fluids such as blood or urine [7,8]. Liquid biopsies can be performed repeatedly with minimal patient discomfort, allowing real-time monitoring of tumour dynamics, assessment of therapeutic response, and detection of minimal residual disease. Importantly, because they capture genetic material shed from multiple tumour sites, liquid biopsies can provide a more comprehensive picture of tumour heterogeneity compared with that obtained from single-site tissue biopsies [4]. Nevertheless, tissue biopsies remain indispensable for initial diagnosis and detailed histopathological characterisation, highlighting that the two approaches are complementary rather than mutually exclusive [9].

Among the various analytes explored for liquid biopsy, RCC-specific urinary microRNAs are especially attractive candidates. miRNAs are small non-coding RNAs of approximately 19–22 nucleotides that regulate gene expression at the post-transcriptional level, influencing a wide range of biological processes including cell proliferation, differentiation, and apoptosis [10,11]. Their aberrant expression has been implicated in the initiation and progression of several malignancies, where they can function either as oncogenes (oncomiRs) or tumour suppressor miRNAs (tumour suppressive miRs) [12].

A key advantage of miRNAs as potential biomarkers lies in their remarkable stability in body fluids, as they are protected from enzymatic degradation by being encapsulated in extracellular vesicles, associated with RNA-binding proteins, or present in highly stable complexes. This stability persists even under adverse conditions such as repeated freeze–thaw cycles or variations in pH and temperature [11,13].

Among the available biofluids, urine represents a particularly attractive source for biomarker discovery in RCC. It is easily and noninvasively obtainable, allows for repeated collections with minimal discomfort to the patient, and provides a direct window into molecular changes occurring in the kidney and urinary tract. The combination of non-invasive sampling, stability of miRNAs, and their ability to reflect tumour-specific alterations makes urinary miRNAs promising candidates for the diagnosis and monitoring of RCC.

Many studies have explored the diagnostic potential of urinary miRNAs, often focusing on the detection of individual candidates, such as *miR-210*, which has been investigated as a promising urinary biomarker for ccRCC [14]. Similarly, the *let-7* family has been detected in urine supernatants of patients with non-metastatic ccRCC, highlighting its potential role in diagnosis [15]. Although other miRNAs such as *miR-122* and *miR-1271* have been reported in association with RCC [16], mainly in tissue-based studies, the present work focused on miRNAs with established biological relevance in RCC and, importantly, with robust and reproducible detectability in urine. Building on these findings, our study was designed to assess a panel of urinary miRNAs rather than a single target, with the aim of improving both sensitivity and specificity in ccRCC detection, thereby refining diagnostic accuracy and reducing the risk of false negatives or false positives associated with individual biomarkers.

## 2. Materials and Methods

### 2.1. Local Ethics Committee and Informed Consent

The study was approved by the Local Ethics Committee—CEAS Umbria—protocol no. 3193/18 on 14 February 2018, and written informed consent was obtained from all participants. All procedures were conducted in accordance with the principles outlined in the Declaration of Helsinki (1975, revised in 2013). Urine collection was performed in compliance with ethical and legal requirements, and all samples and data were analysed anonymously to ensure participant confidentiality.

### 2.2. Study Population and Sample Collection

This study prospectively enrolled 53 patients between October 2023 and August 2025, including samples collected pre-operatively, 5 days after surgery, and 1 month post-intervention. Eleven subjects were recruited by the University of Chieti-Pescara, while the remaining 42 were enrolled at the Urology Clinic of University of Perugia, thereby ensuring a broader representation of the clinical variability.

Inclusion criteria included patients aged over 30 years with renal neoplasia. The exclusion criteria were current or recent (within 30 days) urinary tract infection, presence of urinary lithiasis, history of RCC, and presence or history of other types of cancer. In addition to the inclusion criteria already described, further clinical information was collected from patients to support the study and ensure proper interpretation of the molecular data.

In patients undergoing nephrectomy, urine collection was repeated at multiple time points to capture changes in miRNA expression: (a) before surgery, to assess expression levels in the presence of a renal mass; (b) five days after surgery, to evaluate miRNA levels in the absence of tumour burden; and (c) 1 month after surgery, to monitor expression after both tumour removal and resolution of post-operative inflammation.

Written informed consent was obtained from all participants of the study, before urine collection. For each subject, 20 mL of urine were collected on the day they refer to the Urological Clinic. Urine samples were collected in 50 mL sterile containers and stabilised immediately by adding approximately 1 mL of “Urine Preservative Single Dose Ampules” (Norgen Biotek, Thorold, ON, Canada). Samples were then stored at +4 °C in dedicated refrigerators until transport. All specimens, obtained during routine sampling, were transferred to the Urology Biotechnology Laboratory at the University of Perugia (Italy) for subsequent processing and analysis.

### 2.3. Laboratory Methods

#### 2.3.1. RNA Extraction and Reverse Transcription

Total RNA was extracted from 3 mL of urine using the QIAamp Circulating Nucleic Acid Kit (Qiagen, Hilden, Germany), following the manufacturer’s protocol. Before RNA isolation, 1 μL of *UniSp2* and *UniSp4* RNA spike-in mix (Qiagen) was added to each sample to monitor extraction efficiency and reproducibility. Reverse transcription (RT) of urinary RNA was performed using the miRCURY LNA RT Kit (Qiagen), which includes *UniSp6*, a proprietary synthetic RNA spike-in control used to assess RT technical performance. The RT reaction was carried out under the following conditions: incubation at 42 °C for 60 min, followed by enzyme inactivation at 95 °C for 5 min. The resulting cDNA was stored at −20 °C according to the manufacturer’s instructions.

#### 2.3.2. RT-Quantitative PCR Analysis

Before RT-qPCR analysis, cDNA was thawed and diluted 1:30, and 3 µL of diluted cDNA were combined with 7 µL of Qiagen PCR master mix for amplification. For each sample, three internal controls were included: *UniSp2* and *UniSp4* (Qiagen spike-in controls for monitoring miRNA recovery), and *UniSp6* (Qiagen spike-in used to assess reverse transcription efficiency). Five target urinary miRNAs (*miR-15b*, *miR-16*, *miR-15a*, *miR-210-3p*, and *miR-let-7b*) and the endogenous normalizer (*miR-20a-5p*) were quantified by RT-qPCR in triplicate. In addition, the three internal controls and a no-template negative control (NTC) were run in duplicate. RT-qPCR was performed on a Rotor-Gene Q instrument (Qiagen) using the miRCURY LNA SYBR^®^ Green PCR Kit (Qiagen) following the manufacturer’s protocol. Cycling conditions included an initial denaturation at 95 °C for 2 min, followed by 40 cycles of 95 °C for 10 s and 56 °C for 60 s for denaturation and annealing/extension. A melting curve analysis was then performed by ramping from 60 °C to 95 °C: samples showing abnormal melting curves were considered non-specific or non-amplified.

The relative expression levels of the five miRNAs were quantified using the 2^−ΔΔCt^ method. Briefly, the ΔCt was obtained by subtracting the Ct of the normalizer from the Ct of each target miRNA for each sample. The ΔΔCt was then calculated by comparing the ΔCt of the case samples with that of the reference group (healthy controls). Finally, the relative expression levels were expressed as fold changes (2^−ΔΔCt^), providing a normalised measure of differential miRNA expression. First, normalisation was performed using the endogenous normalizer *miR-20a-5p* and separately using the synthetic spike-in control *SP6*. To minimise technical and biological variability, the mean value of the two normalisation methods was calculated for each sample. All subsequent analyses of the five target urinary miRNAs were then performed on this averaged normalised dataset. After normalisation, a composite parameter named “*miRNA Sum*” was calculated as the sum of the normalised expression levels of all five target miRNAs.

### 2.4. Statistical Analysis

Before performing group comparisons, data distribution was assessed using the Shapiro–Wilk test for each urinary miRNA. The analysis revealed that miRNA expression values deviated from normality across the studied groups (*p* < 0.05 for most miRNAs). Therefore, non-parametric statistical tests were applied for subsequent analyses. Differences in miRNA expression between two independent groups (e.g., healthy controls vs. patients) were analysed using the Mann–Whitney U test, while paired comparisons (e.g., pre- vs. post-operative samples) were evaluated using the Wilcoxon signed-rank test.

For variables that met the assumptions of normality, Student’s *t*-test for independent samples was applied to compare mean expression levels between groups. Correlations between miRNA expression and continuous clinical parameters (e.g., age, tumour size, tumour volume, surgical and ischemia time) were evaluated using Spearman’s rank correlation coefficient. In patients, the association between categorical clinical variables (gender, smoking status, BMI, diabetes, hypertension, chronic renal failure, vascular invasion, collecting system invasion and WHO/ISUP grading for ccRCC and Papillary Tumour) and miRNA expression levels were assessed using the Mann–Whitney U test for binary variables and the Kruskal–Wallis test for ordinal variables.

All statistical analyses were performed using both Excel 2013 and SPSS version 20.0 (IBM, Armonk, NY, USA). Two-tailed *p*-values < 0.05 were considered statistically significant. Data are presented as median (interquartile range, IQR) or mean ± standard deviation (SD), as appropriate.

## 3. Results

The study cohort comprised 46 patients with histologically confirmed renal cell carcinoma (clear cell, papillary, and chromophobe subtypes), 7 patients with benign renal masses, and 47 sex- and age-matched healthy controls. All patients presenting with a renal mass underwent either radical or partial nephrectomy, depending on the clinical features of the lesion. To ensure representativeness, different histological subtypes of renal cancer were included, with ccRCC being the most frequent variant [16]: in our cohort of 53 patients, there were 33 cases of ccRCC, 7 cases of papillary RCC (pRCC), and 6 cases of chromophobe RCC (chRCC). In addition, 7 cases of benign oncocytomas were also included.

Clinical and demographic data were reported in Table 1.

### 3.1. Technical and Quality Controls with Synthetic Spike-in RNAs

To minimise the impact of technical and inter-individual variability on miRNA quantification by RT-qPCR, synthetic non-human spike-in RNAs (Qiagen) were added throughout the workflow following the manufacturer’s instructions. Specifically, *Spike-in 2* and *Spike-in 4* were added during the RNA isolation procedure to monitor extraction efficiency, while *Spike-in 6* was included in each reverse transcription reaction to assess cDNA synthesis performance.

To assess the stability of the exogenous spike-in controls (*SP2*, *SP4*, and *SP6*) across the study population, expression data were first tested for normality using the Shapiro–Wilk test. The results showed that while *SP2* and *SP4* exhibited approximately normal distributions in all groups (*p* > 0.05), *SP6* did not (*p* < 0.05 in most groups), justifying the use of both parametric and non-parametric approaches for the comparative analyses.

For *SP2* and *SP4*, one-way ANOVA tests revealed no statistically significant differences in expression levels among healthy controls, patients with tumour, post-surgery patients, and patients one month after surgery (*SP2*: F = 1.458, *p* = 0.228; *SP4*: F = 1.849, *p* = 0.141).

For *SP6*, the Kruskal–Wallis test was performed, also indicating no statistically significant differences among the four groups (χ^2^(3) = 6.85, *p* = 0.077). All the statistical tests reported above are available in Table S1.

Overall, these results demonstrate that the expression levels of all spike-in controls (*SP2*, *SP4*, and *SP6*) remained consistent across all clinical conditions and time points–pre-surgery, post-surgery, and at one-month after surgery, confirming their stability and reliability as normalisation references in urinary miRNA expression analyses.

### 3.2. Preoperative miRNA Profiles Compared to Healthy Controls

Normality was assessed using the Kolmogorov–Smirnov and Shapiro–Wilk tests for each clinical subgroup. Most distributions significantly deviated from normality (*p* < 0.05), particularly in healthy controls and ccRCC patients, indicating the need for non-parametric statistical analyses. Only a few miRNAs within small histological subgroups (e.g., oncocytoma and chromophobe RCC) displayed approximately normal distributions. Differences in miRNA expression between healthy controls and patients with renal masses (all subtypes) were assessed using the Mann–Whitney U test.

Results showed significantly lower expression of *miR-15b* (U = 927.5, *p* = 0.039) and *miR-16* (U = 903.0, *p* = 0.025) in patients compared to healthy controls. No significant differences were observed for *miR-15a*, *miR-210*, or *miR-let-7b* (*p* > 0.05).

When comparing the overall urinary miRNA expression (*miRNA Sum*) between healthy controls and patients with RCC, a statistically significant difference was observed (U = 752.5, Z = −3.290, *p* = 0.001), indicating a markedly altered expression pattern in patients with cancer (Table S2).

When comparing urinary miRNA expression levels between healthy controls and patients with ccRCC only, significant differences were observed for *miR-15b*, *miR-16*, and *miR-15a*, with lower expression levels in the ccRCC group (Mann–Whitney U = 514.5, *p* = 0.011; U = 487.0, *p* = 0.005; and U = 554.0, *p* = 0.030, respectively). Conversely, *miR-210-3p* and *miR-let-7b* did not show significant differences between groups (*p* = 0.581 and *p* = 0.953, respectively). When considering the overall miRNA expression (*miRNA Sum*), the difference between healthy controls and ccRCC patients remained highly significant (U = 452.5, Z = −3.157, *p* = 0.002), confirming a distinct urinary miRNA signature associated with ccRCC (Table 2).

In addition, a binary logistic regression was performed to evaluate the predictive value of the composite miRNA score (*miRNA Sum*) for distinguishing patients with ccRCC from healthy controls (Table 3).

The binary logistic regression model is expressed as Equation (1):

(1) $$p=\;\frac{1}{1+e^{-\left(\beta 0+\beta 1X1+\ldots +\beta kXk\right)}}$$

where

β_0_ = 0.526 (intercept), and β_1_ = −0.088 (regression coefficient for the miRNA Sum).

The model can therefore be written as follows in Equation (2):

(2) $$p\left(ccRCC\right)=\;\frac{1}{1+e^{-\left(0.526-0.088\;X\;miRNA\;Sum\right)}}$$

This equation represents the model used to estimate the probability that a given sample belongs to the ccRCC group based on the combined expression levels of the five target miRNAs after normalisation (*miRNA Sum*).

**Figure 1:**
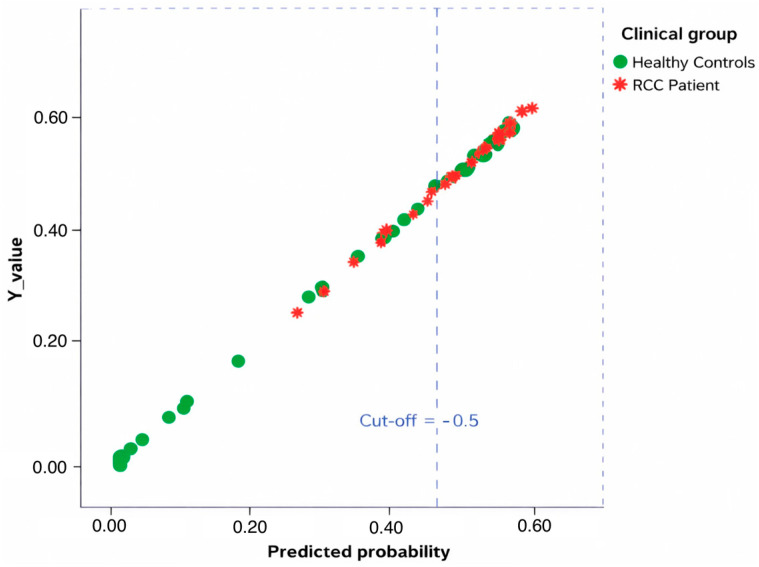
Predicted Probability of Disease Status (Healthy subjects vs. ccRCC patients) with a Cut-off Threshold of 0.5. Each dot represents an individual sample, coloured in green for healthy subjects and red for patients with RCC. The vertical dashed lines indicate the cut-off thresholds at 0.5 used to discriminate between the two clinical groups. The clear separation of most samples across these thresholds highlights the model’s ability to distinguish between healthy and diseased subjects based on urinary miRNA expression profiles (*miRNA Sum*).

The model showed that *miRNA Sum* was a significant predictor of disease status (Figure 1) (B = −0.088, SE = 0.041, Wald = 4.572, *p* = 0.032), with an odds ratio of 0.916 (95% CI: 0.845–0.993), indicating that for each unit increase in *miRNA Sum*, the odds of being classified as ccRCC decreased by approximately 8.4%. The model intercept was not statistically significant (B = 0.526, *p* = 0.144) (Table 3).

**Figure 2:**
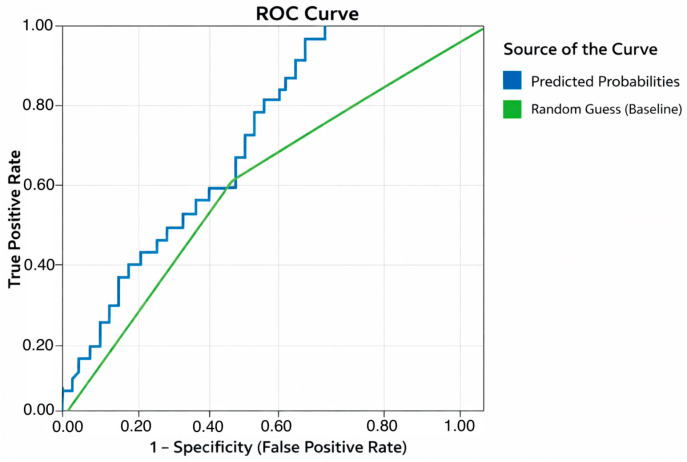
Receiver operating characteristic (ROC) curves for the composite miRNA score in distinguishing ccRCC patients from healthy controls. The ROC analysis of predicted probabilities (blue line in the figure) from the logistic regression model yielded an area under the curve (AUC) of 0.708, indicating moderate discriminative ability.

Receiver Operating Characteristic (ROC) analysis of the predicted probabilities from the logistic regression showed an area under the curve (AUC) of 0.708, indicating moderate discriminative ability of the *miRNA Sum* for differentiating ccRCC patients from healthy controls (Figure 2). When using the predicted group classification, the AUC was 0.625. Some ties between positive and negative cases were present, which may slightly bias the statistics. Overall, these results suggest that the composite miRNA score is a relevant biomarker with potential clinical utility in identifying ccRCC.

### 3.3. Post-Nephrectomy miRNA Dynamics

In the comparison between healthy subjects and all patients one month after surgery (Table 4), none of the urinary miRNAs analysed showed statistically significant differences in expression levels (Mann–Whitney U test, *p* > 0.05 for all comparisons). However, *miR-16* exhibited a trend toward higher variability between groups (*p* = 0.076), suggesting a possible modulatory trend that may require confirmation in a larger cohort.

When restricting the analysis to patients with ccRCC only, the results were consistent, with no significant differences detected compared to healthy controls. Nonetheless, *miR-16* again showed the lowest *p*-value (*p* = 0.053), indicating a borderline trend that may reflect disease-specific alterations in urinary miRNA expression following surgery.

To further investigate surgery-related changes in miRNA expression, paired pre- and post-operative samples were analysed. Shapiro–Wilk test indicated that all miRNA expression values deviated significantly from a normal distribution (*p* < 0.001 for all; Shapiro–Wilk *p* = 0.000 for all), justifying the use of non-parametric analyses. Therefore, the Wilcoxon signed-rank test was applied to compare pre- and post-surgery expression levels in these paired samples.

Results showed that *miR-16*, *miR-15a*, *miR-210*, and the overall *miRNA Sum* exhibited significant differences between the pre- and post-operative conditions (Z = −2.243, *p* = 0.025; Z = −3.064, *p* = 0.002; Z = −2.171, *p* = 0.030; and Z = −3.082, *p* = 0.002, respectively). In contrast, *miR-15b* and *miR-let-7b* did not show statistically significant changes after surgery (Z = −1.235, *p* = 0.217; Z = −0.785, *p* = 0.432, respectively) (Table 5).

These results suggest that the expression levels of specific urinary miRNAs, particularly *miR-15a*, *miR-16*, and *miR-210*, tend to decrease after tumour removal, indicating a potential link between their expression and tumour presence [14].

Subsequently, miRNA expression was compared between the pre-operative samples and those collected at 1 month post-surgery. The normality of the differences in miRNA expression between the pre-operative and 1-month post-operative time points was assessed using both the Kolmogorov–Smirnov and Shapiro–Wilk tests. All comparisons revealed a significant deviation from normality (Kolmogorov–Smirnov: 0.202–0.376, *p* ≤ 0.01; Shapiro–Wilk: 0.460–0.886, *p* ≤ 0.009), indicating that non-parametric tests were appropriate.

Accordingly, paired differences were analysed using the Wilcoxon signed-rank test. The results showed that *miR-15b* (Z = −1.561, *p* = 0.119), *miR-16* (Z = −1.238, *p* = 0.216), and *miR-15a* (Z = −1.238, *p* = 0.216) did not significantly change at 1 month post-surgery. *miR-210* showed a trend toward decrease (Z = −1.870, *p* = 0.061), while *miR-let-7b* was slightly reduced (Z = −1.964, *p* = 0.049). Overall, the *miRNA Sum* was significantly lower one month after surgery than before surgery (Z = −2.085, *p* = 0.037) (Table 6).

Finally, changes in urinary miRNA expression levels between the 5-day and 1-month post-surgery time points were evaluated using the Wilcoxon signed-rank test. The analysis revealed that *miR-15a* exhibited a statistically significant change (Z = −2.375, *p* = 0.018), whereas *miR-15b* (Z = −0.093, *p* = 0.926), *miR-16* (Z = −1.232, *p* = 0.218), *miR-210* (Z = 0.000, *p* = 1.000), *miR-let-7b* (Z = −1.606, *p* = 0.108), and the overall *miRNA Sum* (Z = −1.605, *p* = 0.108) did not show significant changes (Table 7).

### 3.4. Correlation Between Urinary miRNA Expression and Clinical Parameters

Spearman correlation analyses were performed to evaluate associations between urinary miRNA expression levels and continuous clinical parameters (age, tumour volume and size, ischemia time, surgical time), whereas the Mann–Whitney U test was used for binary variables in our cohort of patients.

All miRNAs showed significant positive correlations with each other, with the strongest associations observed between *miR-15b* and *miR-16* (ρ = 0.844, *p* < 0.001) and between *miR-15a* and *miR-16* (ρ = 0.775, *p* < 0.001). *miR-210* was also positively correlated with the other miRNAs (ρ = 0.371 to 0.389, *p* < 0.01), whereas *miR-let7b* showed weaker and mostly non-significant correlations with other miRNAs. As expected, the individual miRNAs were highly correlated with each other and with the *miRNA Sum* (all *p* < 0.05), confirming the consistency of the urinary miRNA measurements across the miRNA panel.

None of the urinary miRNAs displayed a significant correlation with tumour volume and tumour size, indicating that urinary miRNA expression levels are independent of tumour size and volume (Table S3).

Spearman’s rank correlation analysis was performed to explore the associations between urinary miRNA expression levels and clinical variables in ccRCC patients. A strong positive correlation was observed among *miR-15b*, *miR-16*, and *miR-15a* (ρ = 0.66–0.84, *p* < 0.001), suggesting a possible co-regulation within the same biological pathways. *miR-210* also showed moderate correlations with these miRNAs (ρ ≈ 0.37–0.39, *p* < 0.01), indicating that its expression pattern is consistent with the overall miRNA profile.

Among clinical parameters, *miR-15b* and *miR-15a* showed a weak but significant positive correlation with age (ρ = 0.33, *p* = 0.016; ρ = 0.27, *p* = 0.049, respectively). Surgical time correlated negatively with *miR-15a* (ρ = −0.32, *p* = 0.039) and *miR-210* (ρ = −0.45, *p* = 0.003), whereas ischemia time showed positive correlations with *miR-16* (ρ = 0.32, *p* = 0.041) and *miR-let-7b* (ρ = 0.33, *p* = 0.036) (Table S3).

We further analysed variables with more than two categories, namely smoking status (non-smoker, former smoker, current smoker) and BMI (normal weight, overweight, type I obesity), using the Kruskal–Wallis H test. Smoking status was significantly associated with several miRNAs: *miR-15b*, *miR-16*, *miR-15a*, and *miR-210* showed significant differences across smoking categories (*p* = 0.009, 0.036, 0.041, and 0.037, respectively), whereas *miR-let-7b* did not differ significantly (*p* = 0.927). The combined *miRNA Sum* also differed significantly among smoking groups (*p* = 0.013). (Table S4) In contrast, BMI categories did not show any significant association with miRNA expression (all *p* > 0.05) (Table S4). 

For binary variables (gender, diabetes, hypertension, chronic renal failure, vascular invasion, and collecting system invasion) no significant differences were observed for most miRNAs across these variables (Mann-Whitney U test, all *p* > 0.05). Specifically, gender did not significantly influence miRNA levels, although *miR-15a* and *miR-let-7b* showed a trend toward higher expression in one gender (*p* = 0.068 and *p* = 0.069, respectively). Similarly, diabetes, hypertension, chronic renal failure, vascular invasion and collecting system invasion were not associated with significant changes in miRNA expression.

To explore the relationship with tumour grading, the association between urinary miRNA expression (individual miRNAs and *miRNA Sum*) and WHO/ISUP grade was analysed in ccRCC and papillary RCC using the Mann–Whitney U test, by grouping grades 1–2 and 3–4 due to the very limited number of WHO/ISUP grade 3–4 cases. This analysis did not show statistically significant differences (all *p* > 0.005) (Table S5).

## 4. Discussion

In the search for reliable non-invasive biomarkers for ccRCC, urinary microRNAs have attracted increasing attention over the past decade. Their stability in urine, combined with their role in tumour biology, makes them particularly attractive candidates for early detection strategies. One of the earliest attempts to explore the potential of urinary microRNAs was carried out by Cochetti and colleagues in 2020 [17]. In that study, the authors began with a bioinformatic screening of publicly available datasets to identify deregulated miRNAs in renal cancer tissues. From this analysis, three miRNAs—*miR-122*, *miR-1271*, and *miR-15b*—emerged as particularly relevant in renal tumour studies. The novelty of the work, however, was not only the tissue-based discovery but also the validation of these candidates in urine samples from patients with ccRCC compared to those of healthy individuals.

To increase diagnostic accuracy, the authors combined the expression data of the three miRNAs into a single composite score, the so-called “7p-urinary score”. The score discriminated patients from controls with an AUC of 0.96, achieving 100% sensitivity and 86% specificity. Such performance suggested that a simple urine test, based on a panel of selected miRNAs, could have real potential as a non-invasive diagnostic tool for ccRCC. While limited by the number of cases, this work provided the first compelling evidence that urinary miRNAs can reflect the presence of a renal tumour with high diagnostic potential.

Recognising the need for validation in a larger population, the same group extended their investigation in a subsequent study published in 2022 [18]. This time, they analysed urine samples from 28 patients with ccRCC and 28 healthy, age- and sex-matched controls. Once again, the expression of the three candidate miRNAs was found to be consistently elevated in patients, confirming the biological plausibility of their role as tumour-associated signals. Importantly, the 7p-urinary score was reapplied in this independent cohort, achieving an AUC of 0.81. Sensitivity remained extremely high (96%), which is particularly valuable since a diagnostic test with a low rate of false negatives could reduce the risk of missed cancer diagnosis. On the other hand, specificity decreased to 65%, meaning that a higher number of healthy individuals were incorrectly classified as positive. Nonetheless, the findings confirmed that urinary miRNAs, particularly when combined into multi-marker panels, can detect renal cancer-associated molecular alterations with good accuracy.

These findings highlight a common issue in translational biomarker research, where promising results from pilot studies often show reduced diagnostic performance in larger validation cohorts, emphasising the need for standardisation and multicentre replication. In urinary miRNA research, this challenge is largely driven by substantial methodological heterogeneity across studies, including differences in patient selection, sample handling, RNA extraction, and miRNA quantification protocols [16]. However, the most critical source of variability is the normalisation strategy adopted for data analysis, which remains a major obstacle to cross-study comparability [19,20,21]. Selecting a suitable reference miRNA is essential for obtaining reliable relative quantification in RT-qPCR analyses. In blood or urine, small RNAs such as U6 or SNORD48 are often used, but their suitability in urine is questionable due to variable excretion rates and susceptibility to dilution effects [22,23]. Although small nucleolar RNAs or exogenous spike-ins are commonly used for normalisation, endogenous urinary miRNAs with stable expression were also employed, as they better reflect biological variability and sample-specific differences.

This study is a comprehensive prospective investigation into the expression dynamics of selected urinary miRNAs in patients with RCC, comparing them to healthy controls and evaluating longitudinal changes before and after surgical tumour removal. By analysing a cohort of 53 patients and 47 controls, including pre-operative, early post-operative (5 days), and late post-operative (1-month) urine samples, we aimed to define the diagnostic and biological significance of a five-miRNA urinary panel (*miR-15a*, *miR-15b*, *miR-16*, *miR-210-3p*, and *miR-let-7b*), normalised through both endogenous (*miR-20a*) and synthetic (*SP6*) controls. In our study, *miR-20a* was selected as the endogenous normalizer based on previous validation in ccRCC urinary samples [16].

The inclusion of repeated sampling after surgery provides novel insights into how urinary miRNA signatures evolve in response to tumour removal and subsequent physiological recovery. The stability of synthetic spike-in RNAs (*SP2*, *SP4*, and *SP6*) across all experimental groups confirmed the robustness and reproducibility of miRNA extraction and reverse transcription procedures. The absence of significant variability in their Ct values across healthy, pre-operative, and post-operative samples ensured that observed differences in urinary miRNA expression were biological rather than technical in nature.

The comparison of urinary miRNA expression between patients with renal masses and healthy controls revealed a consistent downregulation of *miR-15b* and *miR-16* in the diseased group. These findings are consistent with prior evidence implicating the *miR-15/16* family in the regulation of cell cycle and apoptosis via targeting of BCL2 and other oncogenic pathways [24,25]. The reduced urinary levels in RCC patients may reflect tumour-driven sequestration, altered exosomal release, or systemic suppression of these miRNAs. Interestingly, *miR-15a* showed a similar but less pronounced pattern, reaching statistical significance only in ccRCC, the most prevalent subtype in our cohort. This subtype-specific downregulation suggests that distinct histological variants of RCC may exhibit different miRNA release profiles, potentially reflecting their underlying molecular heterogeneity [26].

When the analysis was restricted to ccRCC cases, significant decreases in *miR-15a*, *miR-15b*, and *miR-16* expression were observed compared to healthy controls, strengthening the diagnostic value of this trio. *MiR-210-3p*, widely known as a hypoxia-inducible miRNA often overexpressed in tumour tissues [27], did not differ significantly in urine between groups. This discrepancy may indicate that urinary *miR-210* levels are influenced by both tumour-related and post-transcriptional factors, potentially diluted by non-tumour renal or systemic sources. Similarly, *miR-let-7b*, a canonical tumour suppressor involved in proliferation and differentiation control [28], exhibited no diagnostic power in this urinary setting.

The combined measure of all five miRNAs (*miRNA Sum*) yielded the most robust discriminatory power. Logistic regression identified the composite miRNA signature as a significant predictor of disease, with an odds ratio of 0.916 and a ROC AUC of 0.708, demonstrating moderate accuracy in distinguishing ccRCC patients from healthy controls, confirming that a multivariate urinary miRNA score can capture disease-associated molecular changes that may not be apparent when evaluating single miRNAs [16,26]. These findings support the potential of using combined miRNA panels as non-invasive biomarkers for RCC detection, consistent with the emerging studies of multiplexed miRNA in the liquid biopsy approach [17,26,29,30].

Longitudinal analyses provided further insights into how urinary miRNA levels evolve following surgical intervention. Within paired samples collected before and five days after nephrectomy, *miR-15a*, *miR-16*, *miR-210*, and the overall *miRNA Sum* were significantly reduced after surgery. This early post-operative decrease likely reflects the removal of tumour-derived miRNA sources and a transient normalisation of urinary molecular profiles once the neoplastic burden is eliminated. Notably, *miR-210* exhibited one of the clearest postoperative declines [31], consistent with reports describing its tumour-associated overexpression and its correlation with hypoxic microenvironments. These data thus reinforce the idea that urinary *miR-210* may serve as a sensitive dynamic biomarker of tumour presence and surgical clearance, as previously proposed in studies of clear cell RCC [32,33,34].

At one month after surgery, most miRNAs remained relatively stable compared with both pre-surgery and early post-surgery samples, suggesting that the urinary miRNA profile re-equilibrates rapidly after tumour removal. However, only *miR-15a* showed significant modulation at one month, while the other miRNAs remained stable. The overall stability of most miRNAs, including the *miRNA Sum*, supports their potential use as reliable non-invasive biomarkers for monitoring RCC patients, indicating that urinary miRNA profiles are robust markers of disease-related molecular changes suitable for longitudinal follow-up. Interestingly, while *miR-210* tended to remain slightly reduced, its borderline statistical behaviour may indicate residual systemic or renal adaptation processes following surgery rather than ongoing tumour activity. Taken together, these observations suggest that most urinary miRNAs tested, including *miR-16* and *miR-let-7b*, achieve a new steady-state profile by one-month post-intervention, consistent with both tissue healing and metabolic normalisation.

Correlation analyses revealed strong inter-miRNA relationships, particularly among *miR-15b*, *miR-16*, and *miR-15a*, underscoring their potential co-regulation within shared molecular networks [26,35]. The positive correlations of *miR-210* with the *miR-15/16* cluster suggest that, despite differing regulatory pathways, these miRNAs may be co-excreted or co-packaged within extracellular vesicles. Importantly, none of the urinary miRNAs correlated with tumour volume or size, indicating that their excretion more closely reflects tumour biology or host systemic responses rather than tumour dimensions [16]. This behaviour enhances their potential as early diagnostic or surveillance biomarkers, even in patients with small renal masses.

The associations between urinary miRNAs and clinical parameters provide additional biological insight into factors influencing miRNA excretion. Weak but statistically significant correlations with age and ischemia time suggest that physiological and procedural variables may exert a modest effect on urinary miRNA levels, without undermining their overall diagnostic reliability. Notably, the inverse correlations observed between *miR-15a* and *miR-210* and surgical time may reflect transient systemic stress or ischemia-related renal responses affecting miRNA release [36,37,38]. In contrast, no significant associations were detected with tumour grade or collecting system invasion. While this may partly reflect limited statistical power—particularly given the small number of cases with calyceal invasion—it is also plausible that the selected urinary miRNAs predominantly capture tumour-related molecular alterations rather than features linked to pathological aggressiveness. These findings underscore the importance of considering demographic and procedural covariates in the translational application of urinary miRNA biomarkers. Overall, although most binary clinical variables did not significantly influence miRNA expression, collecting system invasion and smoking history emerged as factors associated with the modulation of specific miRNAs, suggesting potential biological relevance in the context of renal tumour pathology.

## 5. Conclusions

Our findings support the utility of urinary miRNA profiling as a noninvasive, reproducible, and biologically informative approach for renal cancer diagnostics and monitoring. The consistent downregulation of the *miR-15/16* family in RCC, its partial normalisation post-surgery, and the dynamic behaviour of *miR-210* are a step forward in the study of a disease-associated urinary signature that mirrors tumour biology and therapeutic response. Although the diagnostic accuracy of the *miRNA Sum* is currently moderate, its diagnostic potential is substantial, as multivariate urinary miRNA models are inherently scalable. The progressive identification of additional RCC-associated miRNAs through ongoing research will allow expansion and refinement of the panel, while the integration of miRNAs strongly correlated with specific clinical variables may further enhance diagnostic performance and support the development of more precise and personalised molecular diagnostic approaches for RCC.

## Figures and Tables

**Table 1:** Demographic and clinical characteristics of the study population.

Variable	Healthy Controls (*n* = 47)	ccRCC (*n* = 33)	Chromophobe (*n* = 6)	Papillary (*n* = 7)	Oncocytoma (*n* = 7)
Age years ± S.D. ^2^ (range)	64.94 ± 10.65 (51–93)	62.78 ± 11.16 (39–82)	68.00 ± 11.85 (49–78)	58.50 ± 7.89 (49–71)	72.75 ± 5.56 (69–81)
Gender Female: *n* (%) Male: *n* (%)	25 (53.20) 22 (46.80)	11 (33.30) 22 (67.70)	6 (100.00) 0 (0.00)	3 (42.90) 4 (57.10)	3 (42..90) 4 (57.10)
Smoking status Never: *n* (%) Ex: *n* (%) Current: *n* (%)	29 (61.70) 10 (21.30) 8 (17.00)	13 (48.10) 8 (29.60) 6 (22.20)	3 (60.00) 2 (40.00) 0 (0.00)	5 (83.30) 1 (16.70) 0 (0.00)	3 (42.90) 1 (14.30) 3 (42.90)
BMI category Normal weight: *n* (%) Over-weight: *n* (%) Obesity I: *n* (%)	25 (53.20) 21 (44.70) 1 (2.1)	14 (53.80) 8 (30.80) 4 (15.40)	3 (50.00) 3 (50.00) 0 (0.00)	1 (16.70) 3 (50.00) 2 (33.30)	3 (50.00) 1 (16.70) 2 (33.30)
Diabetes No: *n* (%) Yes: *n* (%)	42 (89.40) 5 (10.6)	19 (67.90) 9 (32.10)	4 (80.00) 1 (20.00)	5 (83.30) 1 (16.70)	3 (75.00) 1 (25.00)
Hypertension No: *n* (%) Yes: *n* (%)	28 (59.60) 19 (40.40)	12 (42.90) 16 (57.10)	2 (33.30) 4 (66.70)	3 (50.00) 3 (50.00)	0 (0.00) 5 (100.00)
CRF ^1^ No: *n* (%) Yes: *n* (%)	47 (100) 0 (0)	24 (88.90) 3 (11.10)	4 (80.00) 1 (20.00)	6 (100.00) 0 (0.00)	4 (100.00) 0 (0.00)
Tumour Volume cc ± S.D. (range)	NA ^3^	89.73 ± 158.11 (0.97 ± 641.16)	95.68 ± 137.87 (7.24–336.26)	28.59 ± 31.09 (2.57–75.92)	4.93 ± 1.75 (3.06–7.16)
Vascular Invasion *n* (%)	NA	5 (15.20)	0 (0.00)	0 (0.00)	1 (14.30)
Collecting System Invasion *n* (%)	NA	13 (39.40)	1 (16.70)	0 (0.00)	0 (0.00)
Partial *n* (%) Radical *n* (%)	NA	21 (77.80) 6 (22.20)	4 (80.00) 1 (20.00)	6 (100.00) 0 (0.00)	4 (100.00) 0 (0.00)
Robotic *n* (%) Open *n* (%)	NA	22 (81.50) 5 (18.50)	4 (80.00) 1 (20.00)	6 (100.00) 0 (0.00)	4 (100.00) 0 (0.00)
Transperitoneal *n* (%) Retroperitoneal *n* (%)	NA	14 (51.90) 13 (48.10)	2 (40.00) 3 (60.00)	2 (33.30) 4 (66.70)	0 (0.00) 4 (100.00)
Surgical Time (min)	NA	167.89	144.00	134.17	123.75
Ischemia Time (min)	NA	11.15	10.00	8.00	10.00

^1^ CRF: Chronic Renal Failure. ^2^ SD: Standard Deviation. ^3^ NA: Not Applicable.

**Table 2:** Mann–Whitney U test between healthy controls and patients with ccRCC, showing significant differences in the expression of *miR-15b*, *miR-16*, *miR-15a*, and *miRNA Sum*.

	*miRNA 15b* Expression	*miRNA 16* Expression	*miRNA 15a* Expression	*miRNA 210* Expression	*miRNA let-7b* Expression	*miRNA * *Sum*
Mann–Whitney U	514.500	487.000	554.000	719.000	769.500	452.500
Wilcoxon W	1075.500	1048.000	1115.000	1280.000	1897.500	1013.500
Z	−2.551	−2.820	−2.165	−0.553	−0.059	−3.157
Asymp. Sig. (2-tailed)	0.011	0.005	0.030	0.581	0.953	0.002

**Table 3:** Binary logistic regression with *miRNA Sum* composite parameter.

	B	S.E.	Wald	df	Sig.	Exp(B)	95% C.I. for Exp(B)	
							Lower	Upper
miRNA Sum	−0.088	0.041	4.572	1	0.032	0.916	0.845	0.993
Constant	0.526	0.360	2.134	1	1.144	1.691		

**Table 4:** miRNA expression in healthy controls compared with 1-month samples of RCC patients.

	miRNA 15b Expression	miRNA 16 Expression	miRNA 15a Expression	miRNA 210 Expression	miRNA let-7b Expression	miRNA Sum
Mann–Whitney U	494.500	437.500	585.500	463.000	475.000	479.000
Wilcoxon W	819.500	762.500	910.500	1591.000	800.000	804.000
Z	−1.100	−1.774	−0.024	−1.473	−1.331	−1.283
Asymp. Sig.	0.271	0.076	0.981	0.141	0.183	0.199

**Table 5:** Wilcoxon signed-rank test applied to pre- and post-surgery paired samples.

	miRNA 15b Expression	miRNA 16 Expression	miRNA 15a Expression	miRNA 210 Expression	miRNA let-7b Expression	miRNA Sum
Z	−1.235	−2.243	−3.064	−2.171	−0.785	−3.082
Asymp. Sig.	0.217	0.025	0.002	0.030	0.432	0.002

**Table 6:** Statistical tests in pre-surgery samples compared to 1-month post-surgery ones.

	miRNA 15b Expression	miRNA 16 Expression	miRNA 15a Expression	miRNA 210 Expression	miRNA let-7b Expression	miRNA Sum
Z	−1.561	−1.238	−1.238	−1.870	−1.964	−2.085
Asymp. Sig. (2-tailed)	0.119	0.216	0.216	0.061	0.049	0.037

**Table 7:** Wilcoxon signed-rank test in 5 days post-surgery and at the 1-month surveillance post-intervention in paired samples.

	miRNA 15b Expression	miRNA 16 Expression	miRNA 15a Expression	miRNA 210 Expression	miRNA let-7b Expression	miRNA Sum
Z	−0.093	−1.232	−2.375	0.000	−1.606	−1.605
Asymp. Sig.	0.926	0.218	0.018	1.000	0.108	0.108

## Data Availability

The datasets generated and analysed during the current study are available from the corresponding author upon reasonable request.

## Supplementary Materials

The following supporting information can be downloaded at: https://www.mdpi.com/article/10.3390/cancers18020285/s1, Table S1: Statistical tests for the exogenous spike-ins technical controls: *SP2*, *SP4*, and *SP6*. Table S2: Statistical tests for comparing miRNA expression in different clinical groups. Table S3: Spearman correlation analysis between the five miRNA expressions and the clinical parameters of RCC patients. Table S4: The Kruskal–Wallis H test comparing the five miRNAs expression with different categorical variables of RCC patients: smoking categories, BMI index. Table S5: Statistical tests comparing the five target miRNAs expression with different binary clinical parameters of RCC patients: gender, diabetes, hypertension, chronic renal failure, vascular invasion, collecting system invasion, and WHO/ISUP grading system.
