# Supplementary material for: Liquid Biopsy in Clear Cell Renal Cell Carcinoma: Diagnostic Potential of Urinary miRNAs
Source: Cancers (Basel). 2026 Jan 16;18(2):285. doi: 10.3390/cancers18020285 (PMC12839065; doi:10.3390/cancers18020285)
Supplement: Supplementary file 1 [file cancers-18-00285-s001.zip › cancers-4050435-supplementary.pdf]

Supplementary file

Table S1. Statistical tests for the exogenous spike-ins technical controls: *SP2, SP4, SP6*.

Controls	Clinical Group	Kolmogorov-Smirnov			Shapiro-Wilk		
		Statistic	df	Sig.	Statistic	df	Sig.
SP2	Healthy Controls	0,051	47	0,200	0,987	47	0,867
	Patient with tumour	0,055	52	0,200	0,982	52	0,636
	Patient post-surgery	0,071	33	0,200	0,973	33	0,565
	Patient 1 month after surgery	0,141	25	0,200	0,926	25	0,069
SP4	Healthy Controls	0,084	47	0,200	0,979	47	0,556
	Patient with tumour	0,085	52	0,200	0,961	52	0,090
	Patient post-surgery	0,153	33	0,050	0,961	33	0,281
	Patient 1 month after surgery	0,136	25	0,200	0,933	25	0,103
SP6	Healthy Controls	0,235	47	0,000	0,852	47	0,000
	Patient with tumour	0,116	52	0,080	0,944	52	0,016
	Patient post-surgery	0,145	33	0,078	0,862	33	0,001
	Patient 1 month after surgery	0,176	25	0,045	0,913	25	0,035

Controls	ANOVA	Sum of Squares	df	Mean Square	F	Sig.
SP2	Between Groups	9,676	3	3,225	1,458	0,228
	Within Groups	338,508	153	2,212		
	Total	348,184	156			
SP4	Between Groups	15,628	3	5,209	1,849	0,141
	Within Groups	431,116	153	2,818		
	Total	446,745	156			

Control	Kruskal Wallis test		
	Chi-Square	df	Asymp. Sig.
SP6	6,852	3	0,077

Table S2. Statistical tests for comparing miRNA expression in different clinical groups.

miRNAs	Clinical Group		Kolmogorov-Smirnov		Shapiro-Wilk
		df	Statistic	Sig.	Statistic
miRNA-15b	Healthy Controls	47	0,368	0,000	0,373
	Oncocytoma	7	0,292	0,072	0,894
	Cromofobe	6	0,172	0,200	0,939
	Papillary	6	0,215	0,200	0,930
	ccRCC	33	0,267	0,000	0,585
miRNA-16	Healthy Controls	47	0,370	0,000	0,388
	Oncocytoma	7	0,268	0,138	0,851
	Cromofobe	6	0,174	0,200	0,936
	Papillary	6	0,291	0,123	0,753
	ccRCC	33	0,296	0,000	0,586
miRNA-15a	Healthy Controls	47	0,409	0,000	0,325
	Oncocytoma	7	0,153	0,200	0,979
	Cromofobe	6	0,143	0,200	0,984
	Papillary	6	0,292	0,120	0,804
	ccRCC	33	0,306	0,000	0,633
miRNA-210	Healthy Controls	47	0,336	0,000	0,424
	Oncocytoma	7	0,259	0,169	0,769
	Cromofobe	6	0,270	0,197	0,814
	Papillary	6	0,262	0,200	0,792
	ccRCC	33	0,261	0,000	0,666
miRNA-let7b	Healthy Controls	47	0,311	0,000	0,592
	Oncocytoma	7	0,234	0,200	0,942
	Cromofobe	6	0,425	0,001	0,633
	Papillary	6	0,366	0,012	0,628
	ccRCC	33	0,175	0,011	0,876

miRNAs	Mann-Whitney U	Wilcoxon W	Z	Asymp. Sig. (2-tailed)
miRNA-15b	927,500	2305,500	-2,064	0,039
miRNA-16	903,000	2281,000	-2,236	0,025
miRNA-15a	1075,500	2453,500	-1,027	0,305
miRNA-210	1207,000	2585,000	-0,105	0,916
miRNA-let7b	1181,000	2559,000	-0,287	0,774
miRNA Sum	752,500	2130,500	-3,290	0,001

Table S3. Spearman correlation analysis between the five miRNA expressions and the clinical parameters of RCC patients.

		miRNA 15b	miRNA 16	miRNA 15a	miRNA 210	miRNA let7b	Age	Tumor Size (cm)	Tumor Volume (cc)	Surgical time (min)	Ischemia time (min)
miRNA-15b	Correlation Coefficient	1,000	0,844	0,663	0,389	0,216	0,332	-0,088	-0,103	-0,060	0,255
	Sig. (2-tailed)	.	0,000	0,000	0,004	0,124	0,016	0,535	0,467	0,710	0,108
	N	52	52	52	52	52	52	52	52	41	41
miRNA-16	Correlation Coefficient	0,844	1,000	0,775	0,388	0,244	0,250	-0,101	-0,131	-0,243	0,321
	Sig. (2-tailed)	0,000	.	0,000	0,005	0,081	0,074	0,477	0,355	0,125	0,041
	N	52	52	52	52	52	52	52	52	41	41
miRNA-15a	Correlation Coefficient	0,663	0,775	1,000	0,371	0,259	0,274	-0,123	-0,103	-0,324	0,210
	Sig. (2-tailed)	0,000	0,000	.	0,007	0,064	0,049	0,385	0,470	0,039	0,189
	N	52	52	52	52	52	52	52	52	41	41
miRNA-210	Correlation Coefficient	0,389	0,388	0,371	1,000	0,255	0,010	-0,162	-0,218	-0,446	0,252
	Sig. (2-tailed)	0,004	0,005	0,007	.	0,068	0,944	0,251	0,121	0,003	0,111
	N	52	52	52	52	52	52	52	52	41	41
miRNA-let7b	Correlation Coefficient	0,216	0,244	0,259	0,255	1,000	0,022	0,103	0,082	-0,141	0,328
	Sig. (2-tailed)	0,124	0,081	0,064	0,068	.	0,879	0,467	0,565	0,379	0,036
	N	52	52	52	52	52	52	52	52	41	41
Age	Correlation Coefficient	0,332	0,250	0,274	0,010	0,022	1,000	-0,069	-0,085	-0,096	-0,119
	Sig. (2-tailed)	0,016	0,074	0,049	0,944	0,879	.	0,625	0,547	0,544	0,452
	N	52	52	52	52	52	53	52	53	42	42
Tumor Size (cm)	Correlation Coefficient	-0,088	-0,101	-0,123	-0,162	0,082	-0,069	1,000	0,952	0,441	-0,024
	Sig. (2-tailed)	0,535	0,477	0,385	0,251	0,565	0,625	.	<0,001	0,004	0,884
	N	52	52	52	52	52	52	52	52	52	52
Tumor Volume (cc)	Correlation Coefficient	-0,103	-0,131	-0,103	-0,218	0,082	-0,085	0,952	1,000	0,398	-0,001
	Sig. (2-tailed)	0,467	0,355	0,470	0,121	0,565	0,547	<0,001	.	0,009	0,997
	N	52	52	52	52	52	53	52	53	42	42
Surgical time (min)	Correlation Coefficient	-0,060	-0,243	-0,324	-0,446	-0,141	-0,096	0,441	0,398	1,000	-0,055
	Sig. (2-tailed)	0,710	0,125	0,039	0,003	0,379	0,544	<0,004	0,009	.	0,728
	N	41	41	41	41	41	42	41	42	42	42
Ischemia time (min)	Correlation Coefficient	0,255	0,321	0,210	0,252	0,328	-0,119	-0,024	-0,001	-0,055	1,000
	Sig. (2-tailed)	0,108	0,041	0,189	0,111	0,036	0,452	0,884	0,997	0,728	.
	N	41	41	41	41	41	41	41	41	41	4

Table S4. The Kruskal-Wallis H test comparing the five miRNAs expression with different categorical variables of RCC patients: smoking categories, BMI index.

	Statistical Test	miRNA-15b	miRNA-16	miRNA-15a	miRNA-210	miRNA-let7b	miRNA Sum
Smoking	Chi-Square	9,448	6,637	6,393	6,599	0,151	8,740
	df	2	2	2	2	2	2
	Asymp. Sig.	0,009	0,036	0,041	0,037	0,937	0,013
BMI	Chi-Square	0,224	1,159	4,791	1,027	2,264	2,349
	df	2	2	2	2	2	2
	Asymp. Sig.	0,894	0,560	0,091	0,598	0,322	0,309

Table S5. Statistical tests comparing the five target miRNAs expression with different binary clinical parameters of RCC patients: gender, diabetes, hypertension, chronic renal failure, vascular invasion, collecting system invasion, and WHO/ISUP grading system.

	Statistical Test	miRNA-15b	miRNA-16	miRNA-15a	miRNA-210	miRNA-let7b	miRNA Sum
Gender (M or F)	Mann-Whitney U	257,000	246,500	231,500	304,500	232,000	300,000
	Wilcoxon W	722,00	711,500	696,500	769,500	485,000	765,000
	Z	-1,352	-1,547	-1,825	-0,473	-1,815	-0,556
	Asymp. Sig. (2-tailed)	0,176	0,122	0,068	0,636	0,069	0,578
Diabetes	Mann-Whitney U	175,000	140,000	152,500	137,000	161,000	138,000
	Wilcoxon W	253,000	218,000	230,500	215,000	239,000	216,00
	Z	-0,139	-1,114	-0,766	-1,199	-0,529	-1,169
	Asymp. Sig. (2-tailed)	0,889	0,265	0,444	0,230	0,597	0,242
	Exact Sig. ($2^{\text{1-tailed Sig.}}$)	0,902[a]	0,276[a]	0,449[a]	0,240[a]	0,611[a]	0,252[a]
Hypertension	Mann-Whitney U	148,000	171,500	205,500	195,000	185,500	211,000
	Wilcoxon W	284,000	307,500	341,500	601,000	321,500	617,000
	Z	-1,855	-1,281	-0,451	-0,708	-0,939	-0,317
	Asymp. Sig. (2-tailed)	0,064	0,200	0,652	0,479	0,348	0,751
Chronic Renal Failure	Mann-Whitney U	69,000	73,500	67,500	36,000	70,000	46,000
	Wilcoxon W	772,000	83,500	770,500	46,000	80,000	56,000
	Z	-0,220	-0,022	-0,286	-1,672	-0,176	-1,230
	Asymp. Sig. (2-tailed)	0,826	0,982	0,775	0,095	0,860	0,219
	Exact Sig. ($2^{\text{1-tailed Sig.}}$)	0,849[a]	0,983[a]	0,783[a]	0,101[a]	0,882[a]	0,237[a]
Vascular Invasion	Mann-Whitney U	117,500	117,500	114,00	100,00	126,000	133,500
	Wilcoxon W	138,500	138,500	135,000	121,000	1207,000	1214,500
	Z	-0,587	-0,587	-0,687	-1,089	-0,344	-0,129
	Asymp. Sig. (2-tailed)	0,557	0,557	0,492	0,276	0,731	0,897
	Exact Sig. ($2^{\text{1-tailed Sig.}}$)	0,566[a]	0,566[a]	0,510[a]	0,291[a]	0,748[a]	0,900[a]
Collecting System Invasion	Mann-Whitney U	93,000	97,500	84,500	59,000	87,500	72,000
	Wilcoxon W	121,000	125,500	112,500	87,000	115,000	100,000
	Z	0,088	0,286	-0,286	-1,410	-0,154	-0,837
	Asymp. Sig. (2-tailed)	0,949	0,780	0,780	0,169	0,880	0,424
Grading WHO/IUSP	Mann-Whitney U	98,000	92,500	112,000	113,500	87,500	106,000
	Wilcoxon W	126,000	120,500	140,000	141,500	115,500	134,000
	Z	0,479	0,228	1,117	1,189	0,000	0,843
	Asymp. Sig. (2-tailed)	0,656	0,824	0,281	0,242	1,000	0,420

[a] Not corrected for ties.
